# A Cross-Sectional Evaluation of the Food Environment at an Australian University Campus

**DOI:** 10.3390/nu15071623

**Published:** 2023-03-27

**Authors:** Daisy H. Coyle, Laura Sanavio, Eden Barrett, Liping Huang, Kristy K. Law, Pabasha Nanayakkara, Jonathan M. Hodgson, Merita O’Connell, Belinda Meggitt, Carrie Tsai, Simone Pettigrew, Jason H. Y. Wu

**Affiliations:** 1 The George Institute for Global Health, The University of New South Wales, Sydney 2050, Australia; 2 School of Public Health, University of California, Berkeley, 200 Centennial Drive Berkeley, Berkeley, CA 94720, USA; 3 School of Medicine, The University of New South Wales, Sydney 2052, Australia; 4 Institute for Nutrition Research, School of Medical and Health Sciences, Edith Cowan University, Perth 6027, Australia; 5 Medical School, University of Western Australia, Perth 6009, Australia; 6 Health Promotions Unit, The University of New South Wales, Sydney 2052, Australia; 7 School of Dentistry, Faculty of Medicine and Health, The University of New South Wales, Sydney 2052, Australia; 8 Sydney Dental Hospital and Oral Health Services, Sydney Local Health District, Sydney 2010, Australia

**Keywords:** university, food environment, nutrition, health star rating, sugar-sweetened beverages, vending machines

## Abstract

University food environments have a strong influence on the dietary choices of students and staff. The aim of this study was to assess the food environment at a large university in Sydney, Australia. Data were collected between March and July 2022 from 27 fixed food outlets and 24 vending machines. The healthiness of the food environment was evaluated using the *Healthy Food and Drink in NSW Health Facilities for Staff and Visitors Framework* (‘Framework’), which assesses food environment parameters including the availability, placement, and promotion of ‘Everyday’ (healthy) and ‘Occasional’ (less healthy) products. Each parameter was evaluated overall and across each food outlet type. Across all outlets, Everyday foods and drinks made up 43.9% of all products. Only two outlets met the Framework’s product availability benchmark of ≥75% Everyday foods and drinks. A total of 43 outlets (84.3%) sold sugary drinks as part of their product range. Occasional products made up 68.4%, 53.3%, and 59.9% of all items for sale at checkout areas, countertops, and eye-level shelves, respectively. Finally, 79.7% of meal deals included Occasional products. Our findings highlight the need to improve the availability, placement, and promotion of foods and drinks sold at a major university campus in Sydney, Australia.

## 1. Introduction

Unhealthy diets, high in sodium, added sugars, saturated and trans fats, and low in fruits, vegetables, and whole grains, are a major modifiable risk factor for the development of non-communicable diseases (NCDs), including type 2 diabetes, cardiovascular disease, and cancer [1,2,3]. In Australia, NCDs currently account for 89% of all deaths and pose a substantial strain on the healthcare system [4,5,6]. There is growing recognition that unhealthy dietary behaviors are shaped by unhealthy food environments. These environments largely feature the widespread availability and discounting of nutrient-poor, energy-dense foods, which nudge consumers towards less healthy choices [7,8].

There is increasing acknowledgement that public institutions, including universities, schools, and health facilities, should provide leadership in providing a healthy food environment to ensure that staff, students, and visitors are not nudged toward unhealthy foods [9,10]. Cultivating healthy food environments is particularly important in the university sector and may help shape accepted norms around healthy food environments, given the recognized role of universities as leading institutions for education and health. Despite this, prior literature has globally demonstrated that university food environments are frequently obesogenic, dominated by unhealthy foods with very limited healthy food options [11,12,13,14]. Unhealthy food environments at universities are especially concerning because the majority of students are young adults who are particularly vulnerable to poor dietary intakes due to lack of disposable income, limited cooking skills, lack of food knowledge, and limited access to kitchen facilities, particularly in shared housing and on-campus accommodations [11,15,16,17].

One strategy to increase the healthiness of institutional food environments is to set nutrition standards or guidelines for the types of foods and drinks that retailers should preferentially procure, market, and sell [9]. While there has been significant development and implementation of nutrition standards for settings, such as schools and healthcare facilities [18,19,20], universities have so far lagged behind.

University management at the University of New South Wales (UNSW), a major Australian university located in Sydney, has committed to improving the campus food environment. This has been driven by the UNSW Good Food Project team, who is in the process of developing and implementing principles around food availability, placement, and promotion. These guidelines will be largely adopted from the *Healthy Food and Drink in NSW Health Facilities for Staff and Visitors Framework* (hereafter, ‘the Framework’). This Framework has been successfully implemented in public hospitals in NSW [21,22] and includes key elements including (i) increasing the availability of healthy foods and drinks, (ii) setting portion size limits for unhealthy foods, (iii) removing unhealthy foods in highly prominent areas (e.g., checkouts), and (iv) limiting meal deals to healthy products only [18,23]. To inform the implementation of the Good Food Project, a clear understanding of the UNSW food environment was required. Therefore, we conducted an objective, baseline assessment of all foods and drinks sold across all food outlets and vending machines at UNSW and compared findings against the Framework.

## 2. Materials and Methods

### 2.1. Study Site

This cross-sectional study took place at the main campus of UNSW, which is located in Kensington, Sydney. UNSW is a large urban university with a population of approximately ~62,000 enrolled students and ~6700 academic and professional staff [24,25].

### 2.2. NSW Health Facilities for Staff and Visitors Framework

The healthiness of all food outlets and vending machines across the UNSW Kensington campus was captured, analyzed, and compared against the *Healthy Food and Drink in NSW Health Facilities for Staff and Visitors Framework* [18]. This Framework was developed and published by the NSW Ministry of Health in 2017 and provides a set of best-practice guidelines for NSW health facilities to improve their retail food environments. The Framework applies to food outlets where foods and drinks are available for purchase for staff and visitors in NSW health facilities.

As displayed in Supplementary Table S1, the Framework classifies foods and beverages into those that are ‘Everyday’ vs. ‘Occasional’.

- Everyday Items: Meals, snacks, and drinks made from products within the five core food groups of the Australian Dietary Guidelines [26], including vegetables, fruit, grains, lean meat, poultry, and dairy. Examples include sandwiches, soups, pasta dishes, yoghurt, and fruit.
- Occasional Items: Foods and drinks mostly high in saturated fat, sugars, and/or salt, often with little nutritional value [18]. They are not needed as part of a healthy diet and should be eaten seldomly, and in small amounts. Examples include pies, hot chips, confectionary, and cakes and pastries.

The Framework also sets a definition for sugary drinks, that is—“sugary drinks are drinks with no nutritional value and which have any sugars added during processing (this excludes milk drinks)” [18]. Examples of sugary drinks include soft drinks, some flavored waters, fruit drinks, cordials, iced teas, energy drinks, and sports drinks.

The Framework includes a target for all food outlets to offer at least 75% Everyday items and no more than 25% Occasional items and to not sell sugary drinks. Foods and drinks brought from home and food and drinks provided as part of fundraising activities are not within the scope of the Framework. The Framework also provides guidance regarding portion size limits and quality of ingredients and sets marketing benchmarks stipulating that promotional activities and placement of foods in prominent positions should be reserved for Everyday foods and drinks [18]. The Framework uses the Health Star Rating (HSR), which is Australia’s voluntary front-of-pack labelling system for packaged foods, to guide food outlet vendors towards healthier versions of packaged foods and drinks [27]. The HSR calculates the overall nutritional profile of packaged foods and drinks, providing a rating from 0.5 to 5 stars in half-star increments; higher stars indicate a healthier choice within a product category. In the Framework, an HSR of 3.5 stars or above is an indicator of a healthier option, and such products should be preferentially stocked and offered by retailers [27].

### 2.3. Data Collection

Over a five-month period, March to July 2022, student volunteers from UNSW were recruited and received training to take photos of all foods and drinks available for sale in each fixed food outlet and vending machine on campus. Volunteers were also instructed to take photographs of menus, product signage, promotions, and/or meal deals at each outlet where available. Training was provided to volunteers by a study staff member to ensure they understood the key Framework parameters and the types of photos required to assess each of these parameters and to ensure that consistent and high-quality photos were taken by all volunteer data collectors. Photographs of each outlet were taken at one point in time.

Photos of each outlet were visually inspected to extract data for analyses. Using previously described methods [28], a member of the study team (LS) extracted information about each food and drink available for sale, including product type (food or drink), product name, brand, and product size/volume. Information was also captured about whether the product displayed an HSR (yes/no) and, if yes, the HSR as reported on pack. Information was also extracted regarding the use of marketing or promotional tactics within or around the outlet. A member of the study team (LS) then categorized each product as either Everyday or Occasional according to the Framework, following previously published assumptions as described in Supplementary Table S1. A second member of the team checked the assigned product classification (Everyday versus Occasional) with any discrepancies resolved by a third member of the team [28].

### 2.4. Study Outcomes

Collected data were collated and analyzed against the key food environment parameters in the Framework as outlined below:

#### 2.4.1. Product Availability

The number and proportion (%) of Everyday products across all food and drink products available for sale were calculated across each outlet type. A food outlet was considered to meet the benchmark if at least 75% of all products available for sale were Everyday products. A second assessed outcome for product availability was the number and proportion of sugary drinks (as a proportion of all drinks) available within each food outlet. The results were presented overall and across each of the main food outlet types to explore potential differences by food outlet.

#### 2.4.2. Portion Size and Ingredients Compliance

As per the Framework, Everyday and Occasional foods and drinks were classified as compliant or non-compliant with regard to both portion size and ingredients [18]. For portion size, limits apply to some Everyday items and all Occasional foods, and these limits vary according to the type of product (e.g., cakes/muffins have a maximum portion limit of 80 g, whereas confectionery has a limit of 50 g). Everyday and Occasional items were considered to also be non-compliant if they contained ‘do not use’ ingredients. These ingredients included palm and coconut oil, animal fats, butter, cream, and nut and chocolate spreads with an HSR <3.5 stars. Assumptions made regarding ‘do not use’ ingredients are displayed in Supplementary Table S1. All sugary drinks were also considered as Occasional and non-compliant. Using this information, all food and drink items were classified under one of the following categories:

- Compliant Everyday Foods and Drinks: Everyday items that met the maximum portion size and ingredient guidelines.
- Compliant Occasional Foods and Drinks: Occasional items that met the maximum portion size and ingredient guidelines.
- Non-Compliant Everyday Foods and Drinks: Everyday items that either (a) exceeded maximal portion size limits; (b) contained ‘do not use’ ingredients; or (c) exceeded both maximal portion size limits AND contained ‘do not use’ ingredients.
- Non-Compliant Occasional Foods and Drinks: Occasional items that either (a) exceeded maximal portion size limits; (b) contained ‘do not use’ ingredients; (c) were a sugary drink; (d) exceeded both maximal portion size limits AND contained ‘do not use’ ingredients; or (e) exceeded both maximal portion size limits AND were a sugary drink.

#### 2.4.3. Nutritional Quality

The nutritional quality of all packaged foods and drinks was assessed according to the HSR. The primary outcome for nutritional quality included the number and proportion of products that had an HSR ≥ 3.5. Since the HSR is implemented on a voluntary basis (i.e., it is not mandatory for products to display this information) [29], we obtained HSR values using two methods: (1) from the front-of-pack where the HSR was available on the product and (2) extracted from the 2021 FoodSwitch database where the HSR was not provided on the product. The FoodSwitch database is a large nutrient composition database that contains nutrition information for ~25,000 packaged food and beverage products that were available for sale across Australian supermarkets during the months of August to November 2021. Information related to data collection, processing, quality assurance, and HSR estimation has been published previously [29,30]. The nutritional quality of each outlet type was assessed according to the mean (SD) HSR.

#### 2.4.4. Marketing

The use of marketing tactics across all food outlets was explored across three key areas: (1) product placement, (2) advertisements, and (3) meal deals (i.e., where food and/or drink products are bundled together for better dollar value, e.g., buy-one-get-one free) [18]. Product placement, checkout areas, countertops, eye-level shelves, and the number and proportion of these locations that displayed Occasional products were assessed. In terms of advertisements, the number and proportion of advertisements that contained Occasional products were calculated overall and across each of the food outlet types. Similarly, the number and proportion of meal deals containing Occasional foods were calculated overall and across each outlet type.

### 2.5. Statistical Analysis

Data were collected and analyzed according to the Framework parameters as described above. All statistical analyses were performed using R Studio (version 1.4.1106) and R (version 4.1.0). A two-sided *p*-value of <0.05 was considered statistically significant.

## 3. Results

### 3.1. Number of Included Outlets

We collected data from a total of 51 food outlets (27 fixed food outlets and 24 vending machines). As outlined in Table 1, this included data from all cafes (*n* = 8/8), all kiosks (*n* = 3/3), the convenience store (*n* = 1/1), 94% of the cafeterias (*n* = 15/16), and 77% of vending machines (*n* = 24/31) on campus [28].

### 3.2. Product Availability

#### 3.2.1. Availability of Everyday Foods

Across all outlets combined, 43.9% of products were classified as Everyday items. When assessed by individual outlet, only 2 of the food outlets (a café and a cafeteria) met the benchmark for ≥75% of all Everyday items (Table 2). Only 6 out of the 46 outlets (all cafeterias) met the ≥75% criteria for foods only, and 6 outlets (all cafés) met the criteria for drinks only (Table 2).

No vending machines, kiosks, or the convenience store met the ≥75% Everyday item benchmark. Across the fixed outlets, cafés had the highest mean proportion of Everyday food and drink items available for sale (61.2%) followed by cafeterias (51.6%) and kiosks (37.7%) (Table 3). In terms of vending machines, drink vending machines had the highest mean proportion of Everyday items available for sale (45.0%) followed by food vending machines (6.7%) and mixed vending machines (4.8%).

#### 3.2.2. Availability of Sugary Drinks

Overall, 43 of the 51 outlets (84.3%) at UNSW sold sugary drinks as part of their product range. The overall proportion of sugary drinks available for sale as a proportion of all drinks across all outlets was 36.7%, which ranged from 15.2% for the kiosks to 45.6% for vending machines (Supplementary Table S2).

### 3.3. Portion Size Limits and Ingredients Compliance

Across all Everyday foods and drinks, only a small proportion (11.7%) were non-compliant due to portion size, ingredients, or both. On the other hand, a substantially greater proportion of Occasional foods and drinks were non-compliant (59.3%), with products often exceeding portion size (especially for Occasional foods, 30.5%) and a large number of Occasional drinks being sugary drinks (65.8%) (Table 4).

### 3.4. Nutritional Quality

A total of 2335 products (605 packaged foods and 1730 packaged drinks) were eligible to display the HSR. Of these eligible products, 8.0% (*n* = 186) had an HSR displayed on the front-of-pack, 83.3% (*n* = 1947) had HSR values obtained from the FoodSwitch database, and 8.7% (*n* = 202) did not have an HSR displayed on the front of package or could not be found in the FoodSwitch database. For products with HSR information (*n* = 2133), 38.2% (*n* = 815) had an HSR ≥ 3.5 (*n* = 42 foods and *n* = 773 drinks). The mean (SD) HSR of foods and drinks across all outlets was 1.6 (1.1) and 2.7 (1.8), respectively. In terms of foods, the mean (SD) HSR was highest for products from kiosks at 3.8 (1.1), followed by cafés at 1.8 (1.1), and the convenience store at 1.6 (1.2) (Table 5). For drinks, the mean (SD) HSR was highest for products from kiosks at 4.0 (1.7), followed by cafés at 3.0 (1.7), and the convenience store at 3.0 (1.9) (Table 5).

### 3.5. Marketing

Across all the food outlets, a large proportion of products available for sale in prominent areas were Occasional foods or drinks, making up 68.4% of all products in checkout areas, 53.3% on countertops, and 59.9% on eye-level shelves (Table 4). In total, 195 advertisements were observed across all outlets, of which 96 (49.2%) promoted Occasional products. Fifty-nine meal deals were observed across all outlets. Of these, 79.7% promoted Occasional products (Table 4). No outlets met the marketing benchmark for the placement of only Everyday products in prominent areas, in meal deals, and as part of advertising and promotional activities.

## 4. Discussion

This study involved a comprehensive evaluation of the healthiness of the fixed food outlets and vending machines available on campus at a large university in Sydney, Australia. The majority of products available for sale were unhealthy Occasional foods that are not recommended as part of a healthy diet as per the Australian Dietary Guidelines [26], and the majority of outlets also sold sugary drinks. Less than half of packaged products available were considered healthy, and most lacked HSR labelling information to indicate their healthiness. There was a high prevalence of the marketing of Occasional foods, with 80% of meal deals promoting Occasional products such as sugary drinks. A key strength of our study is the comprehensive evaluation of multiple food environment parameters that are each critical drivers of consumer dietary choices [18].

The finding that the majority of products currently available for sale at UNSW are unhealthy is consistent with prior research [13,31,32]. For example, an audit conducted in 2019 across 61 vending machines located across three campuses at the University of Newcastle found that 95% of foods and 86% of drinks available for sale were considered unhealthy [31]. Similarly, an audit conducted in 2014 found that 95% of snacks and 49% of drinks for sale in vending machines across a large urban university within NSW were considered unhealthy [13]. Our study builds on and substantially extends these prior investigations by exploring the healthiness of food and drink options at both fixed food outlets and vending machines. In line with this prior research, our current findings suggest that much work is needed to support food retailers and vending machine operators at UNSW to increase the healthiness of their product offerings in line with recommendations [18].

Another novel finding from this study is the considerable number of products that exceeded portion recommendations, especially for Occasional foods and drinks. This was particularly notable for sugary drinks, which is concerning, given their strong associations with dental decay, excess weight gain, and type 2 diabetes [33,34,35,36,37]. Larger portion sizes, particularly of discretionary foods, have increased substantially over the last few decades in Australia and other countries [38,39], and modelling studies suggest that limits to portion sizes of discretionary products can confer significant health benefits [40]. Reducing the portion size of Occasional products across food outlets would not require food retailers to substantially change their product offerings, just to stock the same products in variations that meet portion size recommendations [18].

Our findings suggest that packaged food products available at UNSW are relatively unhealthy and that it is also difficult for students and staff working at the University to identify healthier options due to many products failing to display the HSR. This situation mirrors the broader retail supermarket environment where concerns have been raised about the voluntary nature of the HSR system. A recent evaluation found that the HSR logo is only displayed on 41% of eligible products in Australia and that its use is skewed towards healthier products [29]. Together, this illustrates the impact of suboptimal national labelling policy efforts and the impact that this has on federal, state, and local level institutional policies and nutrition standards that rely on the HSR to set clear recommendations. In the absence of a mandatory HSR system, one practical way to support retailers to stock healthier packaged food products could be to make them aware of existing databases that contain HSR information, such as FoodSwitch [30], which they could use to inform their decision making.

Prior research has demonstrated that products placed at eye-level or in high-traffic positions achieve higher product sales [41,42]. The results of the present study therefore suggest that the current physical layout of food outlets at UNSW likely encourages purchases of unhealthy foods. Prior intervention studies have found that promotional and marketing activities that focus on healthier products do not adversely impact overall sales and profit [43]. For example, an Australian study examined the impact of removing unhealthy drinks from main fridges to out of sight to behind a counter [43]. Over 6 weeks, the proportion of beverage sales represented by unhealthy drinks reduced from 33% to 10%, with a corresponding increase in sales of healthier drinks, resulting in the overall sales volume remaining unchanged [43]. The potential impact on dietary choices of such an intervention in university contexts should be investigated in future studies.

The overall poor food environment quality at universities in Australia [13,31,32] currently including UNSW is not surprising given the relative lack of institutional leadership and policies targeting improved food environments in this setting [22]. This is in contrast with efforts that have been underway in Australia and globally around schools and health facilities [9]. For instance, after the implementation of the *Healthy Food and Drink in NSW Health Facilities for Staff and Visitors Framework*, Everyday food purchases increased by 9% [23], and sugary drinks were removed from all NSW hospitals [18]. Such healthy food environment initiatives also appear to be popular, with 92% of all staff and visitors surveyed indicating their support [21]. Recent survey data suggest university students and staff are similarly supportive of healthy food environment policies and practices [44,45].

While the present findings demonstrate that there is a large gap between an ideal food environment and the current food environment at UNSW, the fact that the UNSW management board has endorsed the UNSW Good Food Project represents progress toward creating a healthier environment for staff and students. The next steps will involve developing a sound and well-resourced implementation plan that supports food retailers in changing the types of foods and drinks they promote and offer to staff and students. Evidence suggests that various elements are needed to support successful implementation of food and nutrition policies in established institutions [9]. These include strong leadership, resources to support retailers implementing policies (e.g., access to databases of compliant vs. non-compliant products), dedicated staff with technical expertise to support retailers achieving compliance, recognition of achievement, integration into institutional policy (e.g., as a requirement of lease), and regular monitoring and evaluation [9].

Our comprehensive and objective evaluation utilized systematic methods to capture, record, and analyze the healthiness of foods and drinks sold across food outlets at a large university. We analyzed the healthiness of the food environment according to *NSW Health Facilities for Staff and Visitors Framework*, allowing a detailed assessment of the types and levels of change required for UNSW to meet established guidelines [18]. Our efforts to extract HSR information from FoodSwitch [30] is an additional strength of this study as it allowed us to conduct a thorough analysis of the HSR of products available for sale at UNSW, despite many of these products not displaying this information on pack.

A limitation of the analyses is that misclassification of products cannot be ruled out. We sought to minimize any misclassification by having multiple researchers independently check data extraction and classification. Additionally, the use of single point data collection likely reduced our ability to capture the full range of product offerings and did not allow us to capture potential seasonal changes in product promotions or availability [8]. However, the overall seasonal impact on our findings is likely low given most of the foods and drinks available for sale were those that are not hugely impacted by seasonality (e.g., packaged foods, pastries, sandwiches, and coffees). Lastly, this study was limited to one university based in NSW, and therefore, the findings may not be applicable more broadly. Further research should look to expand these audits to other universities for both comparability and to increase the representativeness of the results. Moreover, future studies should monitor food retailer progress toward meeting the UNSW Good Food Project goals once the policy is implemented.

## 5. Conclusions

In this comprehensive evaluation of the healthiness of the food environment at a large university campus in Sydney Australia, we found that the majority of food outlets failed to achieve the benchmarks reported in the NSW Healthy Food and Drink Framework. These findings highlight that effective nutrition policies and tools to improve the food environment in the university setting are needed to substantially improve the availability, placement, and promotion of foods and drinks sold in fixed food outlets and vending machines.

## Figures and Tables

**Table 1:** Types and number of food outlets included in the study.

Outlet Type (*n*)	Description
Café (*n* = 8)	A food outlet that sells freshly prepared hot beverages and may sell a selection of pre-made fresh food products such as pastries.
Cafeteria (*n* = 15)	A food outlet that sells either hot or cold fresh food products served to order, may also have a selection of premade foods.
Convenience store (*n* = 1)	A food outlet that sells largely manufacturer packaged products. Limited fresh options available.
Kiosk (*n* = 3)	A food outlet that sells foods and drinks that may be hot, chilled, ambient, fresh or frozen. Usually has limited or no seating for customers. Foods and drinks are mainly not consumed on the premises.
Vending machine (*n* = 24)	Three types: food only, beverage only, mixed food and beverage.

**Table 2:** Number and percentage of outlets meeting the Framework benchmark of ≥75% Everyday food and/or drinks.

Outlet Type (*n*)	≥75% of All Everyday Items *n* (%)	≥75% of Everyday Foods *n* (%)	≥75% of Everyday Drinks *n* (%)
All outlets combined (*n* = 51)	2.0 (3.9)	6.0 (11.8)	6.0 (11.8)
Café (*n* = 8)	1.0 (12.5)	0.0 (0.0)	6.0 (75.0)
Cafeteria (*n* = 15)	1.0 (6.7)	6.0 (40.0)	0.0 (0.0)
Convenience store (*n* = 1)	0.0 (0.0)	0.0 (0.0)	0.0 (0.0)
Kiosk (*n* = 3)	0.0 (0.0)	0.0 (0.0)	0.0 (0.0)
Vending machine (*n* = 24)	0.0 (0.0)	0.0 (0.0)	0.0 (0.0)

**Table 3:** The amount of Everyday foods and drinks available for sale as a mean percentage of all products across each outlet type.

Outlet Type (*n*)	% Everyday Mean (SD)	% Everyday Mean (SD)	% Everyday Mean (SD)
Fixed Outlet	All items combined	Foods	Drinks
Café (*n* = 8)	61.2 (22.2)	28.1 (16.8)	75.4 (23.3)
Cafeteria (*n* = 15)	51.6 (14.5)	62.0 (23.2)	39.8 (17.0)
Convenience store (*n* = 1)	20.6 (0.0)	11.3 (0.0)	43.3 (0.0)
Kiosk (*n* = 3)	37.7 (6.7)	8.3 (14.4)	55.0 (16.7)
Vending machines	All items combined	Foods	Drinks
Drink vending (*n* = 11)	45.0 (14.3)	N/A	45.0 (14.3)
Food vending (*n* = 5)	6.7 (3.7)	6.7 (3.7)	N/A
Mixed vending (*n* = 8)	4.8 (3.9)	6.1 (3.4)	3.2 (6.5)

SD: standard deviation.

**Table 4:** Proportions of products across compliant and non-compliant categories based on portion size, quality of ingredients, and type of product as per the Framework guidelines.

Compliance Categories	Proportion of Products (%)			
	Everyday Foods	Occasional Foods	Everyday Drinks	Occasional Drinks
**Compliant**	88.5	60.3	88.2	24.6
**Non-Compliant**	11.6	39.6	11.8	75.4
- Contains “do not use” ingredients	7.7	10.6	0.7	0.1
- Exceeds portion size limits	2.8	19.9	10.6	9.5
- Exceeds portion size limits and contains “do not use” ingredients	1.1	9.1	0.5	0
- Exceeds portion size limits and is a sugary drink	n/a	n/a	n/a	11.5
- Is a sugary drink	n/a	n/a	n/a	54.3

**Table 5:** The mean (SD) Health Star Rating across food and drinks available for sale, by outlet type.

	Health Star Rating, Mean (SD)	
Outlet Type	Foods	Drinks
Café	1.8 (1.1)	3.0 (1.7)
Cafeteria	1.6 (1.2)	2.5 (1.7)
Convenience store	1.6 (1.2)	3.0 (1.9)
Kiosk	3.8 (1.1)	4.0 (1.7)
Vending machine	1.6 (1.0)	2.7 (1.9)

## Data Availability

The data presented in this study are available on request from the corresponding author. The data are not publicly available given it contains individual-level retail data.

## Supplementary Materials

The following supporting information can be downloaded at: https://www.mdpi.com/article/10.3390/nu15071623/s1, Supplementary Table S1: Summary of assumptions used to inform categorisation of compliant and non-compliant Everyday and Occasional foods; Supplementary Table S2: Total number of sugary drinks available for sale across each outlet type.
