# Supplementary material for: A Cross-Sectional Evaluation of the Food Environment at an Australian University Campus
Source: Nutrients. 2023 Mar 27;15(7):1623. doi: 10.3390/nu15071623 (PMC10097222; doi:10.3390/nu15071623)
Supplement: Supplementary file 1 [file nutrients-15-01623-s001.zip › nutrients-2279008-supplementary.pdf]

Supplementary Table S1. Summary of assumptions used to inform categorisation of compliant and non-compliant Everyday and Occasional foods.

Menu item	Freshly prepared on site/packaged	Assumed category	Reasoning
**Sandwiches/wraps/rolls/toasties**			
Fillings – lean meat, tuna, salmon, roast chicken, reduced-fat cheese, salad/ vegetables, egg, falafel	Freshly prepared/ packaged	**E**	Assume fillings are Everyday and have ≥3.5 Health Star Rating (HSR)
Fillings – bacon, sausages, prosciutto, coated/crumbed meat (e.g., Chicken schnitzel)	Packaged	**O**	Assume fillings are Occasional and have ≥3.5 HSR<br>For portion sizes assume:<br>• Processed meat (e.g., ham, bacon) <60g.<br>• Sausages < max 60g.<br>• Crumbed and coated meat (e.g., chicken tenders) <140g.
**Bread**			
Bread e.g., white, wholemeal, multi-grain, rye	Packaged	**E**	Assume all bread has ≥3.5 HSR therefore Everyday
Margarine on bread	Packaged	**E**	Assume margarine has ≥3.5 HSR
**Sushi and Salads**			
Sushi	Freshly prepared or packaged with lean meat e.g., tuna rolls, teriyaki chicken	**E**	Assume the following fillings are Everyday - Tuna, salmon, fresh chicken breast, avocado, vegetables or tofu.
	Freshly prepared or packaged with crumbed meat	**O**	Assume the following fillings are Occasional - crumbed meats, processed meats<br>**Note:** aligns with salad and processed meat assumption but not covered in guidelines
Salads containing 'non crumbed meat'	Freshly prepared/ packaged	**E**	Assume all salad ingredients are Everyday have ≥3.5 HSR
Salads containing crumbed meat e.g., Chicken tender salad	Freshly prepared/ packaged	**O**	Assume Occasional if salad contains Occasional ingredients such as crumbed or processed meats (≥3.5 HSR).<br>Assume Salad ingredients are Everyday fresh unpackaged or Everyday packaged with ≥3.5 HSR and/or on the 'buy' section of the Essential Shopping List.

Caesar salad	Freshly prepared/ packaged	**E**	Assume Caesar salad contains Everyday packaged ingredients that have ≥3.5 HSR.
**Burgers**			
Burgers (uncoated meat, tofu, fish, beef, chicken, vegetable, lentil, uncrumbed falafel)	Freshly prepared/ packaged	**E**	Assume burger patties are made with Everyday ingredients (lean meat, vegetables or lentils) or packaged ingredients which have ≥3.5 HSR and/or foods from the Essential Shopping List.<br>If packaged burger patty, assume portion size < max
Burgers (crumbed or coated meat/falafel)	Freshly prepared/ packaged	**O**	Assume burger patties has ≥3.5 HSR
**Meat/fish/poultry and alternatives**			
Processed meat e.g., devon, salami, bacon, frankfurters, hot dogs	Packaged	**O**	Check portion size and HSR label on FoodSwitch database
Tuna, salmon, roast beef, skinless chicken, turkey	Packaged	**E**	Assume all lean meats are Everyday and have a ≥3.5 HSR
**Hot food**			
Pasta	Freshly prepared (tomato, mince based or cheese base)	**E**	Assume pasta is made with fresh Everyday ingredients or packaged ingredients which have a ≥3.5 HSR and/or foods from the Essential Shopping List.<br><br>Assume pasta portion size is < max
	Packaged (tomato, mince based or cheese base)	**E**	Check portion size and HSR label on FoodSwitch database
	Freshly prepared with processed meat e.g., carbonara pasta	**O**	Assume occasional items e.g., processed meat have ≥3.5 HSR<br><br>Assume pasta portion size is < max
	Packaged pasta with processed meat	**O**	Check portion size and HSR label on FoodSwitch database
Pizza (does not contain processed meats)	Freshly prepared	**E**	Assume pizza is made on thin crust with fresh unpackaged ingredients or packaged ingredients which have ≥3.5 HSR and/or foods from the Essential Shopping List<br>Assume pizza portion size is < max
	Packaged	**E**	Check portion size and HSR label on FoodSwitch database
Pizza	Freshly prepared	**O**	Assume pizza is made with fresh Everyday ingredients or packaged ingredients which

(contains processed meats)			have ≥3.5 HSR and/or foods from the Essential Shopping List<br>Assume portion size of processed meat is < max portion size, e.g., ham should be <60g<br>Assume pizza portion size is < max
	Packaged	**O**	Check portion size and HSR label on FoodSwitch database
Soup	Freshly prepared	**E**	Assume soup is made with fresh Everyday ingredients or packaged ingredients which have ≥3.5 HSR and/or foods from the Essential Shopping List. Assume lean meats are used.
	Packaged	**E**	Check portion size and HSR label on FoodSwitch database
Jacket Potato	Freshly prepared	**E**	Assume jacket potato is made with fresh Everyday ingredients or packaged ingredients which have ≥3.5 HSR and/or foods from the Essential Shopping List<br>Assume portion < max
Hot chips	Packaged	**O**	Check portion size and HSR label on FoodSwitch database
Garlic bread	Freshly prepared	**O**	Assume appropriate portion size (half English muffin or pita, 1 slice of bread, 75g).<br>Assume garlic bread is made with fresh Everyday ingredients or packaged ingredients which have ≥3.5 HSR and/or foods from the Essential Shopping List
	Packaged	**O**	Check portion size and HSR label on FoodSwitch database
Pies (meat pie, vegetable pie)	Freshly prepared	**O**	If products portion size is <180g for pastry-top pies and <250g if potato-top
Pies (meat pie, vegetable pie)	Freshly prepared	**ONC**	If products portion size is >180g for pastry-top pies and >250g if potato-top OR has a HSR of <3.5 stars
Savoury pastry e.g., Sausage Roll	Freshly prepared	**O**	if savoury pastry portion size < 120g
Savoury pastry e.g., Sausage Roll	Freshly prepared	**ONC**	If savoury pastry portion size > 120g OR has a <3.5 HSR
Hot food products with no ingredient information e.g. This could include but not limited to macaroni and	Freshly prepared	**Assign according to appropriate category in guidelines (E or O)**	If no ingredient list available, assume home-made products are made with fresh Everyday ingredients or packaged ingredients which have ≥3.5 HSR and/or foods from the Essential Shopping List.

cheese, lasagne, meat balls, fried rice, quiche, nachos			Assume portion size requirements are met
	Packaged	**Assign according to appropriate category in guidelines (E or O)**	Check portion size and HSR label on FoodSwitch database
Salads/sandwiches or hot prepared foods	Freshly prepared (with visible Occasional ingredients)	**ONC**	Ingredient list or visible use of :<br>-dressings made from cream, sour cream, crème fraiche<br>-chocolate nut spread and other nut spread <3.5 HSR<br>-sauces made from cream or butter<br>-regular coconut milk and cream
**Snacks**			
Fruit Cup	Freshly prepared	**E**	Assume fruit cup is made with Everyday fresh fruit with no toppings added.
	Packaged	**E**	Check portion size and HSR label on FoodSwitch database
Corn cobette	Freshly prepared/ packaged	**E**	Assume corn cobette is an Everyday vegetable and is not prepared with salt or butter.
Dips - vegetable based, yoghurt	Freshly prepared	**E**	If vegetable based: assume made with fresh Everyday vegetables and/or ingredients from the Essential Shopping List.<br><br>If dairy based: assume products used have a ≥3.5 HSR
	Packaged	**E**	Check HSR label on FoodSwitch database
Dips – cream cheese or sour cream based	Freshly prepared	**ENC**	Assume product has a <3.5 HSR
	Packaged	**ENC**	Check HSR label on FoodSwitch database
Pikelets and pancakes	Freshly prepared	**E**	Assume no toppings are added and/or toppings are from the “Buy” section of the Essentials shopping list and applied according to preparation instructions e.g. thin spread of jam
	Packaged	**E**	Check HSR label on FoodSwitch database
Popcorn	Freshly prepared	**E**	Assume popcorn is air-popped, plain (contains no salt and butter)
	Plain/ packaged	**E**	Check portion size and HSR label on FoodSwitch database, if >50g, then **ONC**
	Flavoured/ microwaved	**O**	Check portion size and HSR label on FoodSwitch database
Salty snacks	Packaged	**O**	Check HSR label on FoodSwitch database
	Packaged, >50g	**ONC**	Over allowed portion size.

	Packaged, with added confectionery (yoghurt coating, choc)	**ONC**	Occasional ingredients added
Muffins	Freshly prepared	**O**	Estimate size, assume no added Occasional ingredients
	Freshly prepared, >80g	**ONC**	Over allowed portion size
	Packaged	**ONC**	Assume ingredients have a HSR of <3.5. Also check size.
Muffin (with added confectionery: choc, icing)	Freshly prepared	**ONC**	Added confectionery<br>Assume choc muffin is <3.5 stars
	Packaged	**ONC**	Assume has <3.5 HSR
Homemade snacks such as cakes, sweet pastries	Freshly prepared	**O**	Assume item meets portion size, if ambiguous, assume ingredients have a HSR of ≥3.5
	Freshly prepared, >80g	**ONC**	Over allowed portion size
	Freshly prepared, with added confectionery: choc, icing	**ONC**	Added confectionery<br>Assume <3.5 stars
Homemade sweet biscuits	Freshly prepared	**O**	Assume item meets portion size, if ambiguous, assume ingredients have a ≥3.5 HSR
	Freshly prepared, >50g	**ONC**	Over allowed portion size
	Freshly prepared, with added confectionery: choc, icing	**ONC**	Added confectionery<br>Assume <3.5 stars
Muesli bars	Packaged	**E**	Check HSR label on FoodSwitch database
	Packaged, >50g	**ENC**	Over allowed portion size
	Packaged, with added confectionery (icing, choc, yoghurt coatings)	**ENC**	Added confectionery<br>Assume <3.5 stars
Confectionery	Packaged	**O**	Assume confectionery is <3.5 stars
	Packaged, if >50g	**ONC**	Over allowed portion size
Jelly	Freshly prepared	**O**	Assume jelly is <3.5 stars
	Packaged	**O**	Assume jelly is <3.5 stars
Jelly sticks, tubes, wobbli or pouch	Confectionary	**O**	Assume sticks, tubes, wobbli or pouch are 'juice jellies therefore confectionary'

are 'juice jellies therefore confectionary'			
**Dairy foods**			
Yoghurt, cheese	Packaged	**E**	Check HSR label on FoodSwitch database
	Packaged, with added confectionery	**O**	Assume all have a <3.5 HSR
**Drinks**			
Water	Packaged	**E**	Assume has a ≥3.5 HSR
Plain milk e.g., cow and plant based milks	Packaged	**E**	Assume plain milk has a ≥3.5 HSR.
Flavoured milk	Freshly prepared	**E**	If ambiguous, assume portion size < max and no more than 1 level tablespoon of milk flavouring or powder e.g., Milo, hot chocolate
	Packaged	**E**	If ambiguous, assume portion size < max and has a HSR of 3.5 stars and above
Milkshakes/ smoothies	Freshly prepared	**E**	If ambiguous, assume portion size < max and is made with ingredients that have a ≥3.5 HSR
	Packaged	**E**	If ambiguous, assume portion size < max and is made with ingredients that have a ≥3.5 HSR
Juice (poppers, pop-tops, frozen juice/ frozen crushed ice drinks)	Packaged	**E**	Check HSR label on FoodSwitch database
Diet soft drinks	Packaged	**O**	Note portion size
**Frozen ice snacks**			
Ice block	Packaged	**O**	Assume portion size <125mL and has a HSR of <3.5 stars
Slushies	Packaged	**E**	If ambiguous, assume 99% fruit juice, portion size < max and HSR of 3.5 stars and above
Ice cream	Packaged	**O**	If ambiguous, assume portion size >125mL and has a HSR of <3.5 stars
Milky bites (frozen milk)	Freshly prepared	**E**	If ambiguous, assume this is low fat flavoured milk with HSR of 3.5 stars and above
Frozen yoghurt	Packaged	**O**	If ambiguous, assume portion size >125mL and has a HSR of 3.5 stars and above.
Ice cups e.g. Made with cordial	Freshly prepared	**O**	If portion size of ice cups > max (125g) and not made with 99% fruit juice.
**Condiments – spreads and sauces**			

Sauces – tomato, BBQ and soy sauce (pg.18)	Packaged	**Compliant**	Assume all sauces are on the ‘buy section’ of the shopping list and soy and satay sauces are salt reduced<br>**Note**: whilst this is the assumption most canteens will not sell salt reduced sauce
Margarine (pg. 17)	Packaged	**Compliant**	Assume margarine has a ≥3.5 HSR.

E: Everyday; ENC: Everyday non-compliant; O: Occasional; ONC: Occasional non-compliant; HSR: Health Star Rating

Supplementary Table S2. Total number of sugary drinks available for sale across each outlet type.

**Outlet type (n)**	**Number of total drinks**	**Number of occasional drinks**	**Number of sugary drinks**	**Sugary drinks as a proportion of total drinks (%)**
All outlets combined (n = 51)	2,409	1,342	884	36.7
Café (n = 8)	413	116	78	18.9
Cafeteria (n = 15)	1,113	670	462	41.5
Convenience store (n = 1)	104	59	47	45.2
Kiosk (n = 3)	191	104	29	15.2
Vending machine (n = 24)	588	393	268	45.6
